# JianPiYiShen formula prevents cisplatin-induced acute kidney injury in mice by improving necroptosis through MAPK pathway

**DOI:** 10.1186/s12906-024-04366-9

**Published:** 2024-02-24

**Authors:** Zhongtang Li, Riming He, Jiahui Liu, Xiaoming Jin, Beibei Jiang, Yunlan Lao, Shudong Yang

**Affiliations:** Department of Nephrology, Shenzhen Traditional Chinese Medicine Hospital, The Fourth Clinical Medical College of Guangzhou University of Chinese Medicine, No.1, Fuhua Road, Futian District, Shenzhen, Guangdong 518033 China

**Keywords:** Acute kidney injury, Cisplatin, JianPiYiShen formula, MAPK pathway, Necroptosis

## Abstract

**Background:**

Acute kidney injury (AKI), characterized by necroptosis and activation of MAPK pathway, causes sudden declines in renal function. To date, efficacious treatments are lacking. JianPiYiShen Formula (JPYSF) has a protective effect on the kidneys. The aim of this study is to explore the mechanism of JPYSF in cisplatin-induced AKI.

**Methods:**

Male C57/BL6J mice were divided into control group, cisplatin group and cisplatin + JPYSF group. Before establishing the model, the cisplatin + JPYSF group was administered JPYSF (18.35 g/kg/day) by gavage for 5 consecutive days. A single intraperitoneal injection of cisplatin (20 mg/kg) was used to establish AKI model. Measurement of renal function and H&E staining were performed to assess renal damage. WB, PCR, TUNEL staining and immunohistochemistry were used to detect related indicators of mitochondrial function, oxidative stress, necroptosis, inflammation and MAPK pathway. And one-way analysis of variance was used to compare group differences.

**Results:**

Compared with the cisplatin group, JPYSF can attenuate AKI, reflected by the decrease in Scr and BUN levels, the improvement of renal tubular injury, and the downregulation of NGAL and KIM1. Cisplatin can induce mitochondrial dysfunction and oxidative stress, triggering necroptosis. In this study, JPYSF improved mitochondrial dysfunction to enhance oxidative stress, as manifested by upregulation of OPA1, PGC-1α, SOD and CAT, and downregulation of DRP1 and MFF. Then JPYSF showed a significant protective effect in necroptosis, as embodied by reduced number of TUNEL-positive cells, decreased the gene expression of RIPK3 and MLKL, as well as downregulation the proteins expression of P-RIPK1, P-RIPK3, and P-MLKL. Moreover, necroptosis can aggravate inflammation. JPYSF ameliorated inflammation by improving inflammatory and anti-inflammatory indexes, including downregulation of TNF-α, IL-6, MCP-1 and LY6G, and upregulation of IL-10. In addition, JPYSF also inhibited MAPK pathway to improve necroptosis by decreasing the expression of P-JNK and P-ERK.

**Conclusion:**

Our data showed that JPYSF prevents cisplatin-induced AKI by improving necroptosis through MAPK pathway, which is related to the improvement of mitochondrial dysfunction, oxidative stress, and inflammation.

**Supplementary Information:**

The online version contains supplementary material available at 10.1186/s12906-024-04366-9.

## Introduction

AKI is a rapid deterioration of renal function characterized by elevated serum creatinine (Scr) levels and reduced urine output. This syndrome can be triggered by various factors such as drug usage, sepsis, ischemia, obstruction, among others [1]. Cisplatin is a commonly used chemotherapy drug in clinical practice. However, its application is limited by severe adverse effects, especially AKI. The incidence of AKI is approximately 30% [2, 3]. To date, no efficacious measures have been developed for clinical prevention or treatment of cisplatin-induced AKI, necessitating further research exploration [3].

Cisplatin accumulated in the renal tubules can give rise to the overproduction of reactive oxygen species (ROS) to induce oxidative stress. This further triggers off various programmed cell deaths, such as necroptosis, apoptosis, pyroptosis, and ferroptosis [2, 3]. Necroptosis plays a significant role in the mechanism of cisplatin-induced AKI [4]. Xu et al. conducted a study demonstrating that the absence of receptor interacting serine/threonine kinase (RIPK)-3 and mixed lineage kinase domain like pseudokinase (MLKL) in mice result in more severe renal tubular damage in cisplatin-induced AKI [5]. In addition, inflammation is a crucial important factor in cisplatin-induced AKI [6]. Tumor necrosis factor (TNF) receptor can trigger the phosphorylation of RIPK1 and RIPK3, recruit MLKL, and form necrosomes, ultimately leading to necroptosis [7]. Research has demonstrated that necroptosis can amplify the generation of inflammatory factors through danger associated molecular patterns (DAMPs), thereby intensifying tissue damage [8]. Moreover, necroinflammation can prompt neutrophil infiltration, further exacerbating the inflammatory response [9]. On the other hand, investigations have revealed that mitochondrial dysfunction and oxidative stress can instigate necroptosis, thereby augmenting its impact [10]. In the pathogenesis of AKI, the disruption of mitochondrial function can lead to an imbalance in the oxidation-antioxidant system, resulting in an augmented generation of ROS and subsequent induction of oxidative stress [11]. Zhang et al. discovered that elevated mitochondrial ROS levels can directly activate the phosphorylation of RIPK1 and RIPK3, thereby initiating the process of necroptosis [12]. The administration of the antioxidant N-acetylcysteine has been shown to effectively mitigate ROS levels and ameliorate renal tubular necroptosis [13].

Mitogen-activated protein kinase (MAPK) pathway, plays a significant role in various kidney diseases, including AKI, renal fibrosis, and diabetic nephropathy [14–16]. A growing body of research indicates that the inhibition of the MAPK pathway can effectively ameliorate necroptosis, as evidenced in diabetic nephropathy, neurological disorders, and osteoarthritis [14, 17, 18]. Specifically, the inhibitor of the mitogen-activated protein kinase P38 alpha (P38) has been found to decrease the expression of RIPK1 and RIPK3 in RGE cells [14]. In their study, François Sipieter et al. discovered that the utilization of extracellular signal-regulated kinases (ERK) inhibitors effectively diminishes the expression of P-MLKL, thereby mitigating tumor necrosis factor-alpha (TNF-α) induced liver cell injury [19]. Furthermore, in a rat model of ischemia-reperfusion (I/R) induced brain injury, the inhibition of mitogen-activated protein kinase 8 (JNK) exhibits the potential to ameliorate necroptosis by downregulating the expression of RIPK3, consequently enhancing brain injury recovery [20].

JPYSF, a Chinese herbal compound, possesses the advantageous characteristics of targeting multiple pathways and targets, thereby demonstrating a protective effect on both acute and chronic kidney injury [21–23]. Prior research has indicated that JPYSF effectively retards the advancement of renal fibrosis in rats with 5/6 nephrectomy or adenine-induced chronic kidney disease (CKD) through the regulation of the mitochondrial control network, enhancement of oxidative stress, and mitigation of inflammation [22, 24]. On the other hand, JPYSF can exert both anti-inflammation and anti-apoptosis via NF-κB pathway to improve cisplatin-induced AKI [23]. To date, however, the potential mechanisms of JPYSF in preventing cisplatin-induced AKI remain to be further studied.

Based on previous evidence, we asked the question of whether JPYSF can prevent cispaltin-induced AKI by improving necroptosis via MAPK pathway. In this study, we established the cisplatin-induced AKI model, and the indicators of necroptosis and MAPK pathway were measured to verify the underlying mechanisms of JPYSF.

## Materials and methods

### Preparation of JPYSF

The preparation of JPYSF was prepared as described [22]. In brief, JPYSF consists of eight herbs, including Astragali Radix, Dioscoreae Rhizoma, Salviae Miltiorirhizae Radix et Rhizoma, Atractylodis Macrocephalae Rhizoma, Cistanches Herba, Amomi Fructus Rotundus, Rhei Radix et Rhizoma, and Glycyrrhizae Radix rt Rhizoma Praeparatacum Melle (Table 1). All herbs were weighed according to Table 1, mixed and boiled in 1200 ml boiled water for 1 h. Then, the supernatant was extracted and dried into lyophilized powder. The powder was stored at -80 °C. Before gavage, the powder was dissolved into distilled water to obtain solution of JPYSF at room temperature.

**Table 1:** Composition of JPYSF

Latin name	English name	Chinese name	Dosage (g)
*Astragalus membranaceus (Fisch.) Bge. var. mongholicus (Bge.) Hsiao*	Astragali Radix	Huang-Qi	30
*Dioscorea opposita Thunb.*	Dioscoreae Rhizoma	Shan-Yao	30
*Salvia miltiorrhiza Bunge.*	Salviae Miltiorirhizae Radix et Rhizoma	Dan-Shen	15
*Atractylodes macrocephala Koidz.*	Atractylodis Macrocephalae Rhizoma	Bai-Zhu	10
*Cistanche deserticola Y. C. Ma*	Cistanches Herba	Rou-Cong-Rong	10
*Amomum kravanh Pierre ex Gagnep.*	Amomi Fructus Rotundus	Dou-Kou	10
*Rheum palmatum L.*	Rhei Radix et Rhizoma	Da-Huang	10
*Glycyrrhiza uralensis Fisch.*	Glycyrrhizae Radix rt Rhizoma Praeparatacum Melle	Zhi-Gan-Cao	6

### Animal experimentation

The animal experiment was conducted in compliance with Experimental Animal Center of Shenzhen TopBiotech Co.Ltd (Shenzhen,China). Approval number is TOP-IACUC-2022-0186. 7-week-old C57/BL6J male mice were purchased from Zhuhai BestTest Bio-Tech Co.Ltd (Zhuhai,China). Mice were adaptively fed in the environment of 20–24 °C and 55% humidity for 7 days. The mice were distributed into three groups (*n* = 6 for each group): (1) control, (2) cisplatin, and (3) cisplatin plus JPYSF (cisplatin + JPYSF). The daily dose of JPYSF for an adult with standard body weight of 60 kg is 121 g. The equivalent dose conversion between mice and humans is 9.1 [25], so the dosage of JPYSF for mice is 9.1 * 121 g/60kg = 18.35 g/kg. Before injecting cisplatin, the cisplatin + JPYSF group received an oral dose of 18.35 g/kg/d JPYSF for a duration of 5 consecutive days [23]. The cisplatin group was given the same amount of distilled water by gavage. The AKI model was established by a single intraperitoneal injection of cisplatin (20 mg/kg; Lot No: P4394; Sigma Aldrich, USA) in the cisplatin group and the cisplatin + JPYSF group [26]. After 72 h, all mice were sacrificed. During this process, mice were subjected to anesthesia using 2.5% Avertin (20 mg/kg; Lot No: T48402; Sigma Aldrich, USA), followed by blood collection from the ocular region. Subsequently, the mice were euthanized through cervical dislocation, and their kidney tissues were extracted. Finally, both the blood and kidney tissues were rapidly frozen in liquid nitrogen and subsequently stored at -80℃ for subsequent analysis.

### Detection of serum creatinine and blood urea nitrogen

Scr and blood urea nitrogen (BUN) were used to evaluate renal function. Blood samples were centrifuged at 3500 rpm for 15 min, and the sera were taken for measurement. Then, levels of Scr and BUN were assessed by enzymatic methods (Hitachi cobas 8000, Roche Diagnostics, Germany).

### Hematoxylin and eosin (H&E) staining and tubular injury score

Paraffin-embedded kidney pieces were cut into 3 μm slices. The sections were dewaxed, rehydrated, and finally stained with H&E according to the procedure. The slices were examined under the microscope (Nikon, Japan). Six randomly selected fields (400X) per mouse were scored for quantification, and the mean was used as the tubular injury score. The Cell lysis, loss of brush border, and cast formation were considered as signs of renal tubular injury. Score according to the proportion of damaged renal tubules: 0, normal; 1, < 10%; 2, 10–25%; 3, 26–75%; 4, > 75% [27].

### Western blot (WB)

WB analyses was performed as described below. Initially, the radio-immunoprecipitation assay (RIPA) lysis buffer (Lot No:89,900; Beyotime, Jiangsu, China), which consisted of protease inhibitors and phosphatase inhibitors (Lot No: P1048; Beyotime, Jiangsu, China), was employed to lyse and extract the proteins present in the kidneys. Subsequently, the protein content was determined using the BCA method (Lot No: 23,227; Thermo Fisher, USA). Equivalent quantities of protein lysates were loaded and subjected to electrophoresis on a 10–12% sodium dodecyl sulfate polyacrylamide gel at a voltage of 100 V. Following this, the protein was transferred to a polyvinylidene fluoride membrane at a voltage of 120 V for a duration of 2 h. To prevent the binding of non-specific proteins, the membrane was treated with 5% non-fatty milk for 1 h at room temperature. Finally, the membrane was divided based on the protein’s kilodalton values. The membranes were subjected to overnight incubation at 4 °C with primary antibodies in order to obtain specific proteins. Subsequently, the membranes underwent a 1-hour incubation at room temperature with secondary antibodies labeled with horseradish peroxidase. Finally, the blot was detected and visualized using Clarity Western Enhanced chemiluminescence (ECL) reagent (Lot No: WBKLS0500; Millipore, USA) on an MP Imager (Bio-Rad, USA). Densitometric analysis of protein bands was conducted using Image-J (Bio-Rad, USA). The primary antibodies used in this experiment were as follows: Anti-NGAL antibody (1:1000, Lot No: ab41105) (Abcam, USA), Anti-SOD1 antibody (1:1000, Lot No: ab308181) (Abcam, USA), Anti-IL-10 antibody (1:1000, Lot No:ab34843) (Abcam, USA), Rabbit Anti-DRP1 (1:1000, Lot No: #8570s) (Cell Signaling Technology, USA), Rabbit Anti-Phospho-SAPK/JNK (1:1000, Lot No: #4668T) (Cell Signaling Technology, USA), Rabbit Anti-Phospho-RIP3 (1:1000, Lot No: #15828T) (Cell Signaling Technology, USA), Rabbit Anti-Phospho-MLKL (1:1000, Lot No: #37,333) (Cell Signaling Technology, USA), Anti-GAPDH (1:2000, Lot No: #2118) (Cell Signaling Technology, USA), Phospho-RIPK1 Monoclonal antibody (1:2000, Lot No:66854-1-Ig) (Proteintech, Wuhan, China), Phospho-ERK1/2 Polyclonal antibody (1:1000, Lot No:28733-1-AP) (Proteintech, Wuhan, China), ERK1/2 Polyclonal antibody (1:2000, Lot No: 11257-1-AP) (Proteintech, Wuhan, China), JNK Monoclonal antibody (1:3000, Lot No: 66210-1-Ig) (Proteintech, Wuhan, China), OPA1 Polyclonal antibody (1:1500, Lot No: 27733-1-AP) (Proteintech, Wuhan, China), MFF Polyclonal antibody (1:1000, Lot No: 17090-1-AP) (Proteintech, Wuhan, China), PGC-1α Monoclonal antibody (1:1000, Lot No:66369-1-Ig) (Proteintech, Wuhan, China), GAPDH Monoclonal antibody (1:5000, Lot No: 60004-1-Ig) (Proteintech, Wuhan, China), Anti-TNF-α (1:300, Lot No: sc-52,746) (Snata Cruz, USA), Anti-IL-6 (1:300, Lot No:sc-57,315) (Snata Cruz, USA), Anti-MCP-1 (1:300, Lot No: sc-52,701) (Snata Cruz, USA), and Mouse TIM-1/KIM-1/HAVCR Antibody (1:500, Lot No: AF1817) (R&D system, USA). MaxVision HRP-polymer anti-mouse/rabbit secondary antibodies (Lot No: KIT-5020; Maixin Biotech, Fujian, China) and Goat Anti-Rabbit IgG secondary antibody (Lot No: #L3012; Signalway, USA) were used in this experiment. The explanation of the original data of WB experiment was as follows. Three different samples per group were used for WB experiment to meet the requirements for biological replication [28]. The membrane was cut according to the kilodaltons of specific proteins. The other parts of the same membrane were also kept. In this study, the original images of all-length cropped blots were presented in the form of supplementary file 3. And the regions of the original blots used in main figures were marked in red boxes.

### Quantitative real-rime reverse transcription-polymerase chain reaction (qRT-PCR)

Total RNA was extricated from kidney samples using the RNA Easy Fast Tissue/Cell Kit (Lot No: DP451; TianGen, Beijing, China) and following the manufacturer’s instructions. RNA was reverse-transcribed into cDNA with the PrimeScript™ RT Master Mix (Lot No: RR036A; Takara, Japan) in accordance with the manufacturer’s protocol. The mRNA levels of SOD2, CAT, RIPK3, MLKL, GAPDH and β-actin were evaluated by the SYBR® Select Master Mix (Lot No: 4,472,908; Applied Biosystems, USA) and ABI QuantStudio 5 Real-Time PCR System (Applied Biosystems, USA) in line with the manufacturer’s instructions. β-actin or GAPDH was used to normalize the expression values of the other genes. Finally, the − 2 ^ΔΔCT^ method was used for analysis. The arrays of the gene-specific primers (TsingKe Biotechnology, Beijing, China) used in this research are presented in Table 2.

**Table 2:** gene-specific primers for qRT-PCR

Gene name	Forward	Reverse
SOD2	GCCCAAACCTATCGTGTCCA	AGGGAACCCTAAATGCTGCC
CAT	GCAGATACCTGTGAACTGTC	GTAGAATGTCCGCACCTGAG
RIPK3	TCTGTCAAGTTATGGCCTACTGG	GGAACACGACTCCGAACCC
MLKL	AATTGTACTCTGGGAAATTGCCA	TCTCCAAGATTCCGTCCACAG
GAPDH	GGCAAATTCAACGGCACAGT	CGCTCCTGGAAGATGGTGAT
β-actin	GGCTGTATTCCCCTCCATCG	CCAGTTGGTAACAATGCCATGT

### Immunohistochemistry (IHC)

Paraffin-embedded kidney slides were processed sequentially through deparaffinization, rehydration, and antigen retrieval. Subsequently, the slides were subjected to a 15-minute incubation with a hydrogen peroxide solution at room temperature, followed by a 1-hour blocking step with 10% goat serum. Subsequently, the slices were incubated overnight at 4 °C with the primary antibody. The following day, the sections were allowed to equilibrate at room temperature for at least 30 min. Subsequently, the sections were treated with diaminobenzidine substrate and counterstained with hematoxylin before being mounted. Finally, the slices were examined under a Nikon microscope (Japan). Three kidney specimens from all groups were blindly caught across three equal microscopic fields (400x) for analysis. With the purpose of reckoning average optical density (AOD), we operated integrated optical density (IOD) measurements on IHC pictures. AOD = IOD / area. After delimiting the zone of interest, the AOD of the chosen precinct (IOD per unit area) was determined utilizing Image-J software. AOD depicts the protein immunoreactivity in the kidneys. The primary antibody used in this experiment was Anti-LY6G antibody (1:100, Lot No: ab25377) (Abcam, USA).

### Terminal dutp nick end-labeling (TUNEL) staining

Necroptosis was detected using a One Step TUNEL Apoptosis Assay Kit (Lot No: C1098; Beyotime, Jiangsu, China) conforming with the manufacturer’s instructions [29]. The renal tissues were paraffin fixed and segmented and then dewaxed. The slices were incubated at room temperature for 25 min with Recombinant Proteinase K. The slices were washed 3 times using phosphate buffered saline (PBS), and then hatched with a TUNEL-staining kit at 37 °C for 1 h. Nuclei were dyed with DAPI after being cleaned 3 times with PBS. Finally, the slices were captured under an optical microscope (Carl Zeiss, Germany). Three kidney specimens from all groups were blindly caught across three equal microscopic fields (400x) in the cortical area for analysis [30]. Blue fluorescence was normal cell nuclei, and green fluorescence was necrotic cell uclei, which were positive cells. The number of TUNEL-positive cells in each slice was determined as the necroptosis index.

### Statistical analysis

All data were analyzed using GraphPad Prism 9.0 (USA). There were at least three independent samples in each group, and one-way analysis of variance was used to compare group differences. All data were expressed as mean ± standard error of the mean, and *P* < 0.05 indicated statistical difference.

## Results

### JPYSF prevents cisplatin-induced AKI in mice

**Fig. 1:**
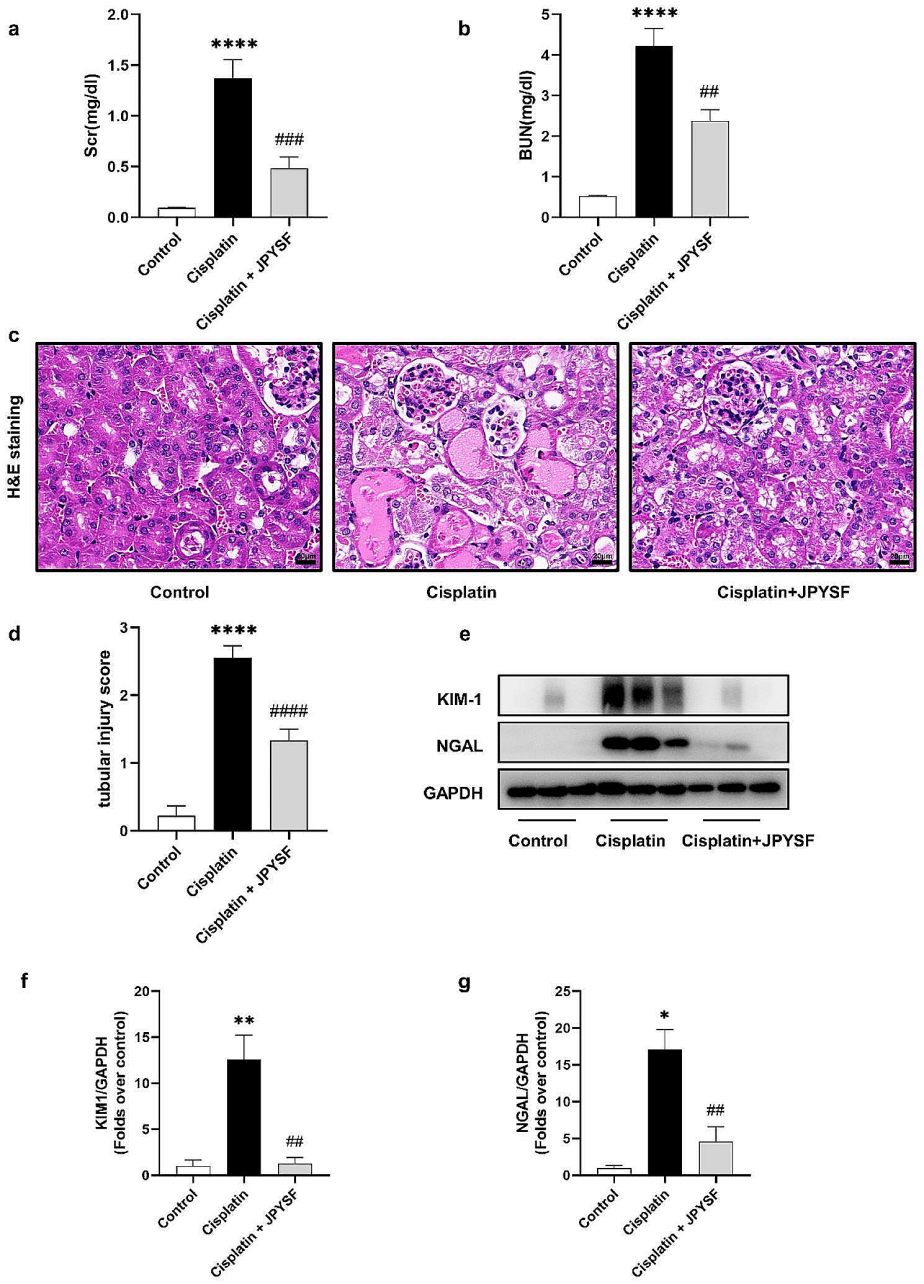
JPYSF prevents cisplatin-induced AKI in mice. (**a**) and (**b**) Levels of Scr and BUN were measured in each group (*n* = 6). (**c**) Representative kidney sections stained for H&E in each mice group. Scale bar = 20 μm. (**d**) represents the semiquantitative analysis of tubular interstitial injury (*n* = 6). (**e**) Representative WB images of KIM-1 and NGAL. (**f**) and (**g**) Densitometric analyses of KIM-1 and NGAL proteins expression normalized to GAPDH content (*n* = 3). Data presented are means ± SEM. ∗*P* < 0.5, ∗∗*P* < 0.01, ∗∗∗∗*P* < 0.0001 vs. the control group; ##*P* < 0.01, ###*P* < 0.001, ####*P* < 0.0001 vs. the cisplatin group

The levels of Scr and BUN were measured, and H&E staining was conducted to evaluate renal damage. Figure 1 demonstrated that cisplatin administration resulted in elevated levels of Scr and BUN (Fig. 1a, b). H&E staining of the cisplatin group revealed significant tubular damage, characterized by cell lysis, loss of brush border, and formation of casts (Fig. 1c). Additionally, the renal tubular injury score was also increased in the cisplatin group (Fig. 1d). Treatment with JPYSF reduced Scr and BUN levels and ameliorated renal tubular injury (Fig. 1a–d). Kidney Injury Molecule-1 (KIM-1) and neutrophil gelatinase-associated lipocalin (NGAL) serve as markers for renal tubular damage. Compared with the cisplatin group, the expression of KIM-1 and NGAL was downregulated by JPYSF (Fig. 1e–g).

### JPYSF improves mitochondiral dysfunction in cisplatin-induced AKI mice

**Fig. 2:**
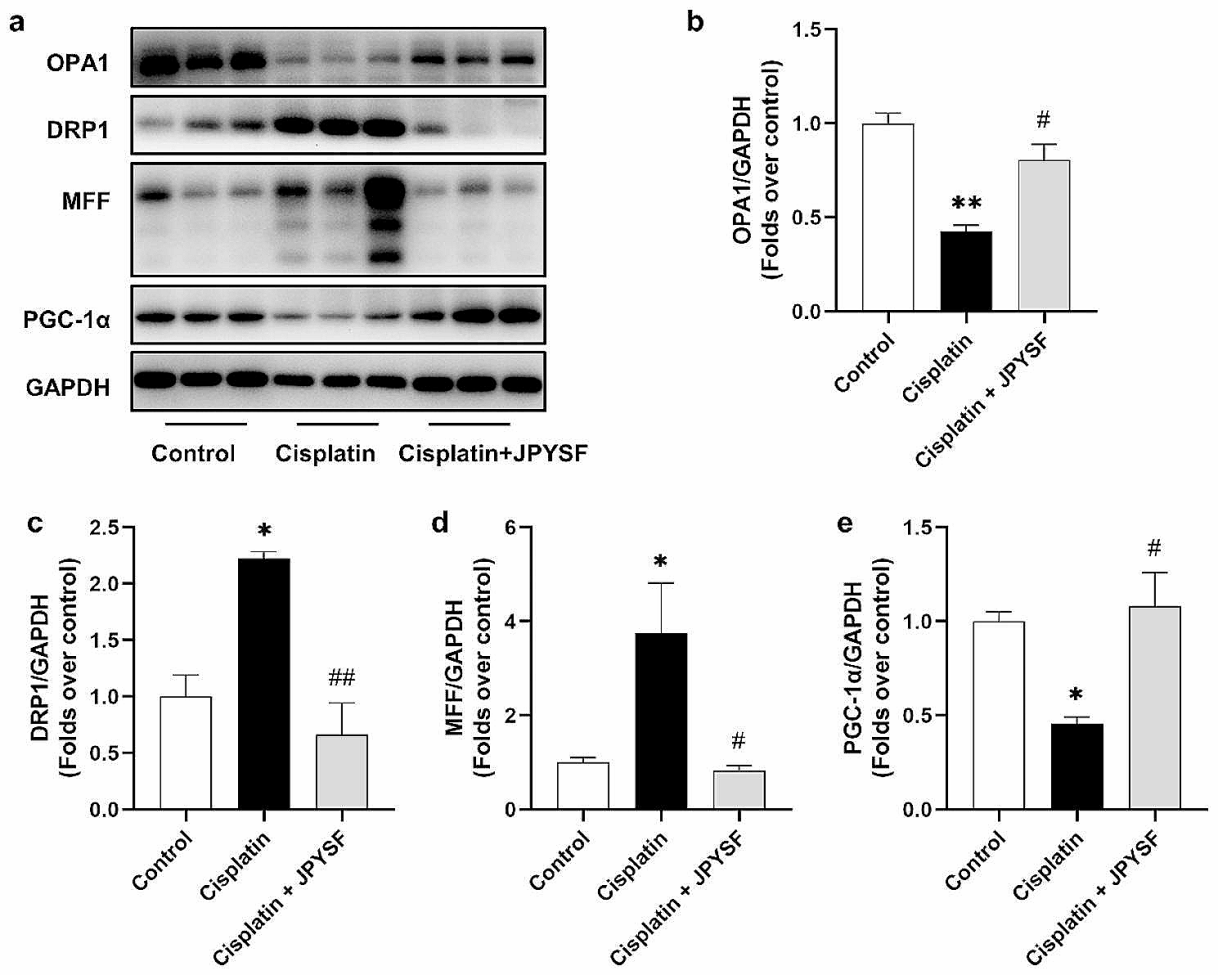
JPYSF improves mitochondiral dysfunction in cisplatin-induced AKI mice. (**a**) Representative WB images of mitochondrial function-associated proteins, such as OPA1, DRP1, MFF and PGC-1α. (**b**) - (**e**) Densitometric analyses of OPA1, DRP1, MFF and PGC-1α proteins expression normalized to GAPDH content (*n* = 3). Data presented are means ± SEM. ∗*P* < 0.5, ∗∗*P* < 0.01 vs. the control group; #*P* < 0.5, ##*P* < 0.01 vs. the cisplatin group

Cisplatin causes mitochondrial dysfunction characterized by disruptions in mitochondrial fission/fusion and mitochondrial biogenesis. The administration of cisplatin disrupted the balance between mitochondrial fusion and fission, resulting in an upregulation of the expression of dynamin-1-like protein (DRP1) and mitochondrial fission factor (MFF), and a downregulation of the expression of optic ptrophy 1 (OPA1) (Fig. 2a–d). Interestingly, the administration of JPYSF was found to ameliorate these effects, upregulating the expression of OPA1 and downregulating the expression of DRP1 and MFF (Fig. 2a–d). Furthermore, cisplatin also impaired mitochondrial biogenesis, as indicated by a decrease in the expression of peroxisome proliferator-activated receptor-γ co-activator 1α (PGC-1α). However, treatment with JPYSF improved mitochondrial biogenesis by increasing the expression of PGC-1α, as compared to the cisplatin group (Fig. 2a, e).

### JPYSF attenuates oxidative stress in cisplatin-induced AKI mice

**Fig. 3:**
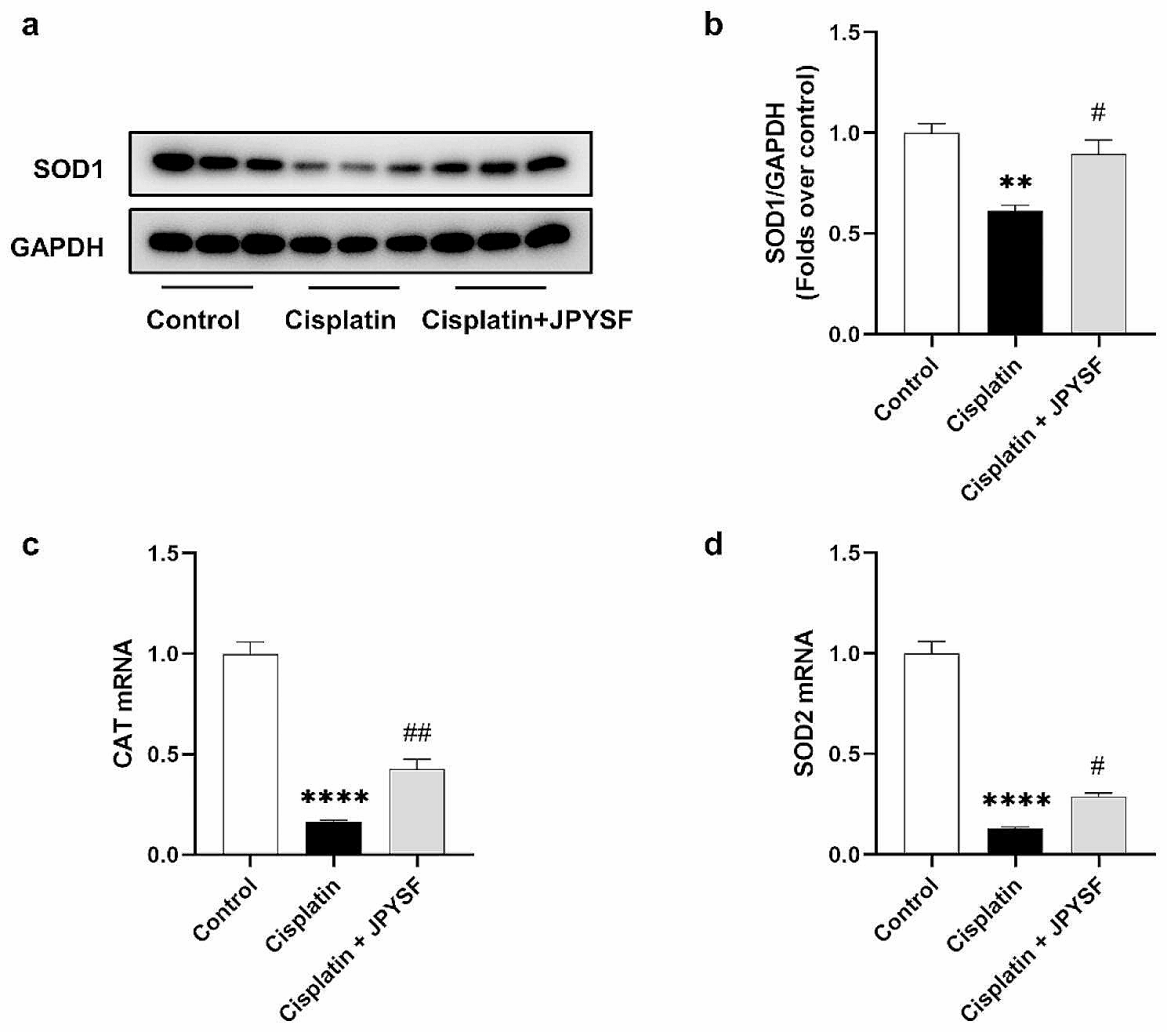
JPYSF attenuates oxidative stress in cisplatin-induced AKI mice. (**a**) Representative WB images of SOD1 in the kidneys. (**b**) Densitometric analyses of SOD1 proteins expression normalized to GAPDH content (*n* = 3). (**c**) and (**d**) mRNA expression levels of CAT and SOD2 in renal tissues were measured by qRT-PCR. Data presented are means ± SEM. ∗∗*P* < 0.01, ∗∗∗∗*P* < 0.0001 vs. the control group; #*P* < 0.5, ##*P* < 0.01 vs. the cisplatin group

The role of oxidative stress in cisplatin-induced AKI is significant. Mitochondrial dysfunction can trigger oxidative stress, thus prompting us to examine the indicators of antioxidant enzymes. The WB results depicted in Fig. 3 demonstrated that cisplatin administration led to a reduction in SOD1 expression. Conversely, the expression of SOD1 was upregulated by JPYSF (Fig. 3a, b). Furthermore, in comparison to the cisplatin group, the mRNA levels of CAT and SOD2 were found to be elevated in the cisplatin + JPYSF group (Fig. 3c, d).

### JPYSF ameliorates necroptosis in cisplatin-induced AKI mice

**Fig. 4:**
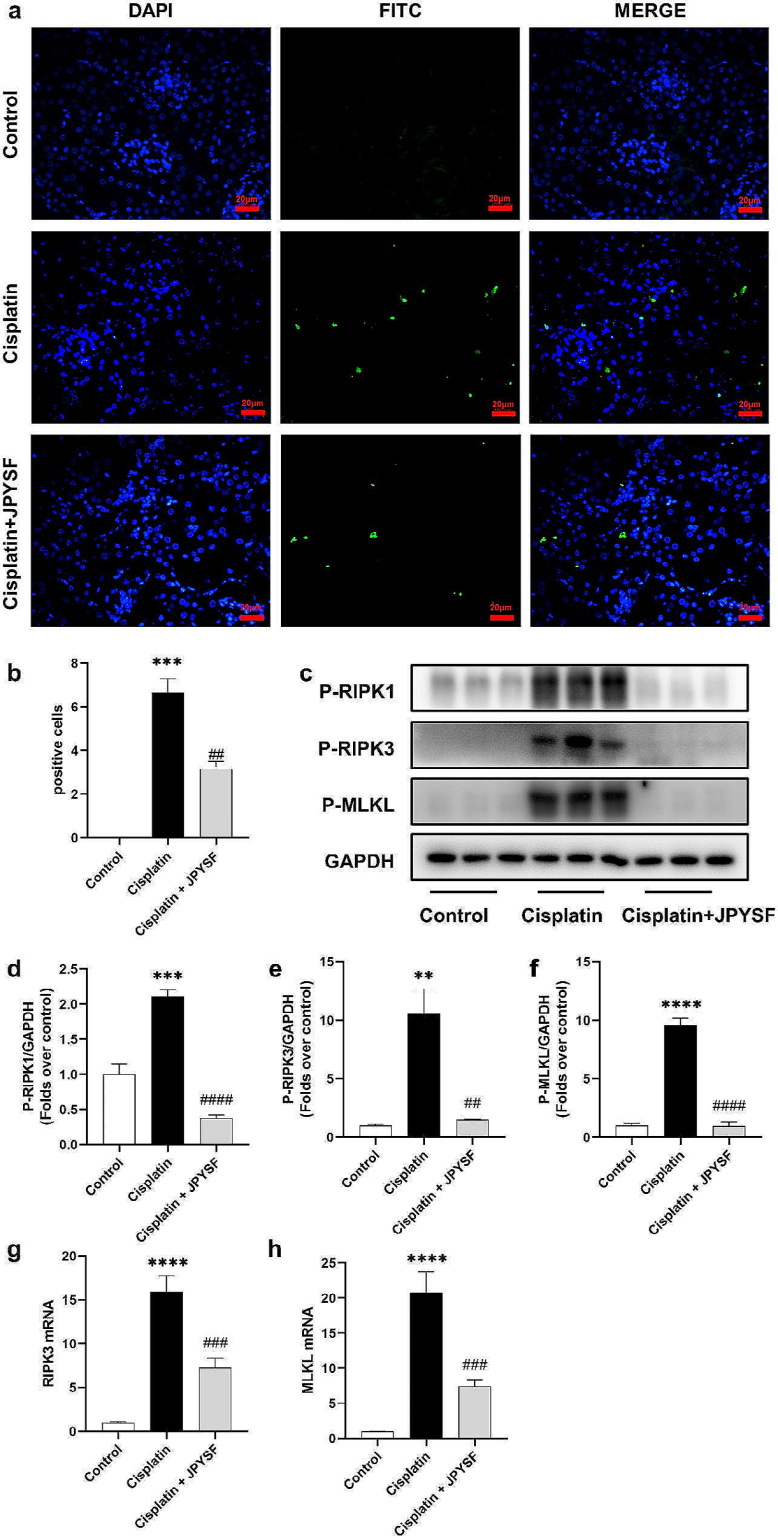
JPYSF ameliorates necroptosis in cisplatin-induced AKI mice. (**a**) Representative images of TUNEL staining in the renal tissues (*n* = 3). Positive cells are visualized as green and normal nuclei are stained with DAPI (blue). (**b**) Quantitative analysis for the number of TUNEL-staining positive cells. (**c**) Representative WB images of necroptotic proteins, such as P-RIPK1, P-RIPK3 and P-MLKL in the kidneys. (**d**) - (**f**) Densitometric analyses of P-RIPK1, P-RIPK3 and P-MLKL proteins expression normalized to GAPDH content (*n* = 3). (**g**) and (**h**) mRNA expression levels of RIPK3 and MLKL in renal tissues were measured by qRT-PCR. Data presented are means ± SEM. ∗∗*P* < 0.01, ∗∗∗*P* < 0.001, ∗∗∗∗*P* < 0.0001 vs. the control group; ##*P* < 0.01, ###*P* < 0.001, ####*P* < 0.0001 vs. the cisplatin group

Necroptosis plays a significant role in the development of cisplatin-induced AKI, with mitochondrial dysfunction and oxidative stress serving as triggers for necroptosis. In this study, we observed that cisplatin administration resulted in mitochondrial dysfunction and oxidative stress. Consequently, we examined biomarkers associated with necroptosis (Figs. 4 and 6). TUNEL staining revealed an increase in the number of renal tubular cell deaths in the cisplatin group, as indicated by the presence of green fluorescence. Notably, treatment with JPYSF significantly reduced the number of TUNEL-positive cells (Fig. 4a, b). Furthermore, we employed WB and PCR techniques to assess necroptosis-related indicators. The WB analysis demonstrated that JPYSF led to a down-regulation of P-RIPK1, P-RIPK3, and P-MLKL expression in comparison to the cisplatin group (Fig. 4c–f). Similarly, the PCR findings indicated an increase in the mRNA levels of RIPK3 and MLKL in the cisplatin group, while a decrease was observed in the cisplatin + JPYSF group (Fig. 4g, h).

### JPYSF suppresses inflammation in cisplatin-induced AKI mice

**Fig. 5:**
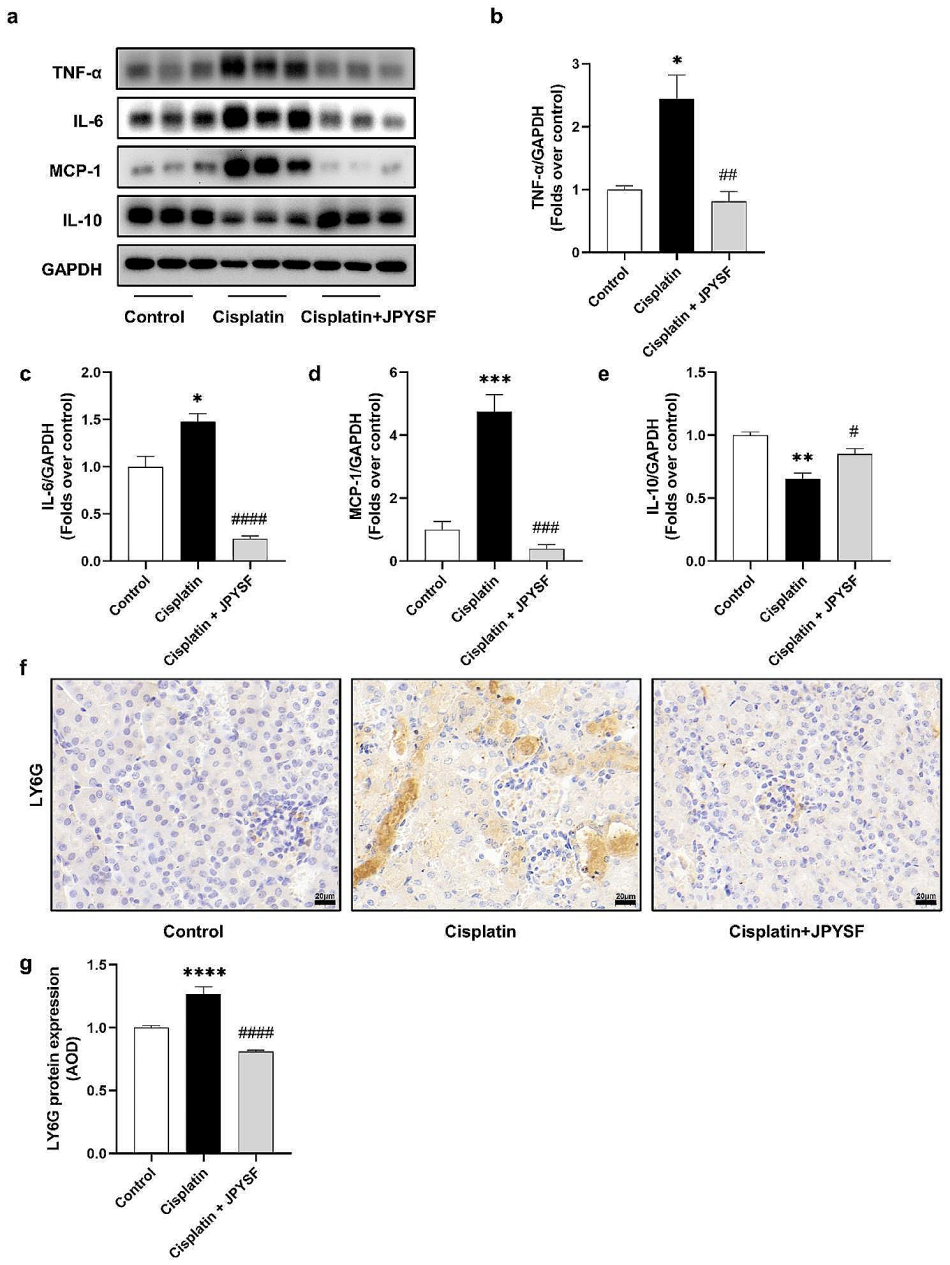
JPYSF suppresses inflammation in cisplatin-induced AKI mice. (**a**) Representative WB images of pro-inflammatory and anti-inflammatory cytokines, such as TNF-α, IL-6, MCP-1, and IL-10 in the kidneys. (**b**) – (**e**) Densitometric analyses of TNF-α, IL-6, MCP-1, and IL-10 proteins expression normalized to GAPDH content (*n* = 3). (**f**) Representative IHC images of LY6G protein in renal tissues (*n* = 3). Scale bar = 20 μm. (**g**) Quantitative analysis of IHC for the LY6G protein expression. AOD = IOD / area. Data presented are means ± SEM. ∗*P* < 0.5, ∗∗*P* < 0.01, ∗∗∗*P* < 0.001, ∗∗∗∗*P* < 0.0001 vs. the control group; #*P* < 0.05, ##*P* < 0.01, ###*P* < 0.001, ####*P* < 0.0001 vs. the cisplatin group

Inflammation is an important manifestation of cisplatin-induced AKI. As shown in Fig. 5, the expression of pro-inflammatory proteins TNF-α, interleukin (IL)-6, and monocyte chemoattractant protein-1(MCP-1) was up-regulated and the expression of anti-inflammatory proteins IL-10 was down-regulated in the cisplatin group. Compared with the cisplatin group, JPYSF decreased the expression of TNF-α, IL-6, and MCP-1 and increased the expression of IL-10 (Fig. 5a–e). On the other hand, neutrophil infiltration can be triggered by inflammation and necroptosis. Lymphocyte antigen 6 complex locus G6D (LY6G) is a marker of neutrophil infiltration. The IHC results showed that the expression of LY6G in the renal tissue increased in the cisplatin group, and JPYSF reduced the expression of LY6G (Fig. 5f, g).

### JPYSF inhibits MAPK pathway in cisplatin-induced AKI mice

**Fig. 6:**
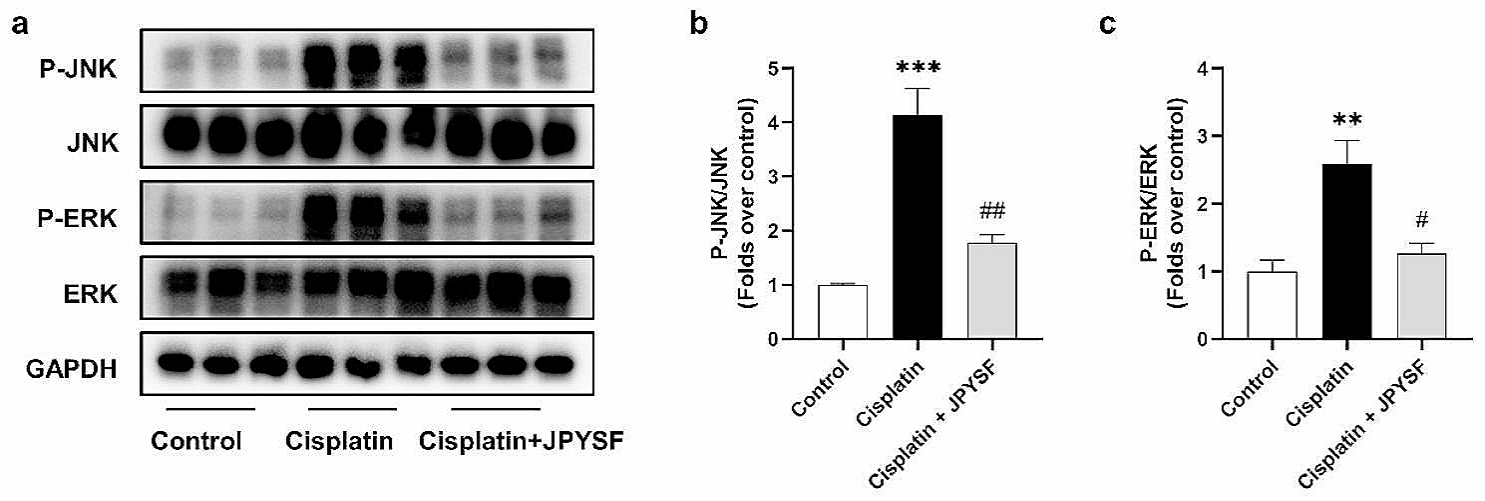
JPYSF inhibits MAPK pathway in cisplatin-induced AKI mice. (**a**) Representative WB images of MAPK pathway, such as P-JNK and P-ERK in the kidneys. (**b**) and (**c**) Densitometric analyses of P-JNK/JNK and P-ERK/ERK proteins expression normalized to GAPDH content (*n* = 3). Data presented are means ± SEM. ∗∗*P* < 0.01, ∗∗∗*P* < 0.001 vs. the control group; #*P* < 0.05, ##*P* < 0.01 vs. the cisplatin group

MAPK pathway plays a crucial role in the pathogenesis of AKI. The WB analysis depicted in Fig. 6 demonstrated an up-regulation of P-JNK and P-ERK expression in the cisplatin group, while a down-regulation was observed in the cisplatin + JPYSF group (Fig. 6a–c). These findings strongly suggested that JPYSF possessed the ability to inhibit MAPK pathway in cisplatin-induced AKI mice.

## Discussion

The present study found that JPYSF prevents cisplatin-induced AKI, embodied by the decrease in Scr and BUN levels and the improvement of renal tubular injury. The renoprotective properties of JPYSF in cisplatin-induced AKI are attributed to its ability to improve necroptosis through MAPK pathway. Furthermore, this novel mechanism of JPYSF may be linked with the improvement of mitochondrial dysfunction, oxidative stress, and inflammation.

Research has provided evidence that cisplatin-induced AKI is characterized by the deposition of cisplatin in renal tubular cells, resulting in mitochondrial damage and subsequent dysfunction [11]. This dysfunction leads to an increase in mitochondrial ROS and the onset of oxidative stress, ultimately triggering the development of necroptosis [10]. Additionally, necroptosis has been identified as a contributor to the exacerbation of inflammatory response through DAMPs [8]. The present study has discovered that JPYSF exhibits renoprotective effects against cisplatin-induced AKI by mitigating necroptosis. This protective effect is associated with the amelioration of mitochondrial dysfunction, reduction of oxidative stress-induced damage, and attenuation of inflammation. In addition, recent findings suggest that the suppression of MAPK pathway can enhance necroptosis [14, 19, 20]. In this particular investigation, it was observed that JPYSF can inhibit MAPK pathway, resulting in the downregulation of P-ERK and P-JNK expression. These results implied that the enhancement of necroptosis by JPYSF may be attributed to its ability to inhibit MAPK pathway.

JPYSF has the potential to ameliorate mitochondrial dysfunction in cisplatin-induced AKI. In the context of cisplatin-induced AKI, cisplatin has been observed to disrupt the balance between mitochondrial fusion and fission, leading to a decrease in OPA1 expression and an increase in DRP1 expression [31]. However, the administration of curcumin has been found to upregulate OPA1 expression and downregulate fission mitochondrial 1 expression, thereby alleviating cisplatin-induced AKI [32]. Consistent with previous research, our findings demonstrated that JPYSF can enhance mitochondrial fusion/fission by increasing OPA1 expression and decreasing DRP1 and MFF expression. Additionally, the perturbation of mitochondrial bioenergetics is also implicated in this disorder [11]. The upregulation of PGC-1α can enhance mitochondrial biogenesis and mitigate apoptosis by reducing mitochondrial ROS production [33]. Liquiritigenin has been found to induce the expression of PGC-1α through NRF2 signaling, thereby improving cisplatin-induced acute tubular injury [34, 35]. Similarly, JPYSF can enhance mitochondrial biogenesis by upregulating the expression of PGC-1α.

JPYSF can exert antioxidant effects in cisplatin-induced AKI. Kim JS et al. found that antioxidant enzymes, such as SOD and CAT, were inhibited in a mouse model of AKI caused by cisplatin. Nonetheless, the levels of SOD and CAT were increased following renal recovery [35]. Quercetin can improve the levels of antioxidant factors such as SOD, CAT, and glutathione peroxidase to improve cisplatin-induced renal tubular damage [36]. In the rats model of CKD, JPYSF attenuates oxidative stress via NRF2 signaling to improve renal fibrosis [24]. In the context of cisplatin-induced AKI, it has been observed that JPYSF exhibits antioxidant properties through the upregulation of SOD1, SOD2, and CAT expressions. Furthermore, cisplatin has the potential to impair the mitochondrial respiratory chain, leading to oxidative stress caused by excessive production of ROS [37]. That suggested that the ability of JPYSF to attenuate oxidative stress may be associated with the improvement of mitochondrial dysfunction.

JPYSF may improve necroptosis and exert anti-inflammatory effect in cisplatin-induced AKI. Cisplatin can stimulate the activation of the RIPK1/RIPK3/MLKL axis, leading to increased necroptosis in renal tubules. Mice lacking RIPK3 or MLKL were protected from cisplatin-induced AKI [5]. 6-shogaol can down-regulate the expression of RIPK1, RIPK3, and MLKL to ameliorate necroptosis in cisplatin-induced AKI [38]. Our data showed that JPYSF can reduce the number of TUNEL-positive cells and down-regulate the expression of necroptosis indicators, such as RIPK1, RIPK3, and MLKL. These findings indicated that JPYSF could improve necroptosis via RIPK1/RIPK3/MLKL axis to prevent cisplatin-induced AKI. On the other hand, studies have found that mitochondrial dysfunction and oxidative stress damage can induce necroptosis [10]. This study showed that JPYSF can improve mitochondrial dysfunction and attenuate oxidative stress damage in cisplatin-induced AKI. This may be the reason why JPYSF can improve necroptosis. In addition, necroptosis can aggravate inflammatory storm through DAMPs to make kidney injury more severe [8]. Xiao et al. found that the inhibitor of necroptosis can reduce renal inflammatory response and improve tubular damage [39]. WB analysis showed that JPYSF can exert anti-inflammatory effect in cisplatin-induced AKI by reducing the expression of pro-inflammatory cytokines, such as IL-6, TNF-α and MCP-1 and increasing the expression of anti-inflammatory factor IL-10. Moreover, necroptosis and inflammation can stimulate neutrophil infiltration, which can exacerbate the production of pro-inflammatory factors [8, 40]. The IHC results showed that an elevation in the expression of LY6G within the cisplatin group, which was subsequently mitigated by the administration of JPYSF. This suggested that JPYSF can improve neutrophil infiltration to diminish renal inflammation.

JPYSF can inhibit MAPK pathway in cisplatin-induced AKI. In I/R-induced AKI, Erythropoietin Receptor/β Common Receptor can mediate MAPK pathway to exert antioxidant, anti-inflammatory, and anti-apoptosis effects to alleviate tubular injury [41]. Hesperetin can attenuate oxidative stress, inflammation and apoptosis by inhibiting MAPK pathway in cisplatin-induced AKI [42]. This study found that JPYSF can improve oxidative stress and inflammation, which may be related to the inhibition of MAPK pathway by JPYSF. Moreover, increasing evidence has demonstrated that MAPK pathway regulates necroptosis. Inhibitors of ERK, P38, and JNK can reduce the expression of necroptosis-related indicators [14, 19, 20]. Zhao et al. found that tea polyphenols can alleviates acetochlor-induced necroptosis via ROS/MAPK/NF-κB signaling in ctenopharyngodon idellus kidney cells [43]. Similarly, selenium can improve necroptosis via P38/JNK/ERK pathway in chicken kidney [44]. In this study, improvement of necroptosis by JPYSF may be related to the inhibition of MAPK pathway.

There are some limitations in this study. Firstly, Cellular experiments should be used to further validate the mechanisms of JPYSF in cisplatin-induced AKI. Secondly, the active ingredients and functions of JPYSF in AKI need to be further studied.

## Conclusion

In conclusion, JPYSF prevents cisplatin-induced AKI by improving necroptosis via MAPK pathway, which is linked with the improvement of mitochondrial dysfunction, reduction of oxidative stress and suppression of inflammation. These findings provide novel insights into the protective mechanisms underlying JPYSF’s action against cisplatin-induced AKI, and suggest its potential as a therapeutic agent.

### Electronic supplementary material

Below is the link to the electronic supplementary material.

**Supplementary Material 1:** The original H&E staining images of JPYSF for tubular injury score

**Supplementary Material 2:** The specific score for each slice in H&E Staining

**Supplementary Material 3:** Western Blot raw data

## Data Availability

The data supporting the conclusions of this manuscript will be made available by the authors, without undue reservation, to any qualified researcher. If you want to get the original data, please contact Dr. Shudong Yang. (E-mail address: shudong_yang@126.com)

## Abbreviations

AKI: acute kidney injury
AOD: average optical density
BUN: blood urea nitrogen
β-actin: beta-actin
CAT: catalase
CKD: chronic kidney disease
DRP1: dynamin-1-like protein
DAMPs: damage-associated molecular patterns
ERK: extracellular signal-regulated kinases
GAPDH: glyceraldehyde-3-phosphate dehydrogenase
H&E: hematoxylin and eosin
IHC: Immunohistochemistry
IL-6: interleukin-6
IL-10: interleukin-10
IOD: integrated optical density
I/R: ischemia/reperfusion
JPYSF: Jian-Pi-Yi-Shen Formula
JNK: mitogen-activated protein kinase 8
KIM-1: kidney injury molecule-1
LY6G: lymphocyte antigen 6 complex locus G6D
MAPK: mitogen-activated protein kinase
MLKL: mixed lineage kinase domain like pseudokinase
MCP-1: monocyte chemoattractant protein-1
MFF: mitochondrial fission factor
NGAL: neutrophil gelatinase-associated lipocalin
OPA1: optic ptrophy 1
PBS: phosphate buffered saline
P-ERK: phospho-extracellular signal-regulated kinases
PGC-1α: peroxisome proliferator-activated receptor-γ co-activator 1α
P-JNK: phospho-mitogen-activated protein kinase 8
P-RIPK1: phospho-receptor interacting serine/threonine kinase 1
P-RIPK3: phospho-receptor interacting serine/threonine kinase 3
P-MLKL: phospho-mixed lineage kinase domain like pseudokinase
P38: mitogen-activated protein kinase P38 alpha
qRT-PCR: quantitative real-time reverse transcription-polymerase chain reaction
ROS: reactive oxygen species
RIPK1: receptor interacting serine/threonine kinase 1
RIPK3: receptor interacting serine/threonine kinase 3
Scr: serum creatinine
SOD: superoxide dismutase
SOD1: superoxide dismutase 1
SOD2: superoxide dismutase 2
TNF: tumor necrosis factor
TNF-α: tumor necrosis factor-alpha
TUNEL: terminal dutp nick end-labeling
WB: western blot
