# Supplementary material for: JianPiYiShen formula prevents cisplatin-induced acute kidney injury in mice by improving necroptosis through MAPK pathway
Source: BMC Complement Med Ther. 2024 Feb 24;24:101. doi: 10.1186/s12906-024-04366-9 (PMC10893720; doi:10.1186/s12906-024-04366-9)
Supplement: Supplementary file 1 — Supplementary Material 1: The original H&E staining images of JPYSF for tubular injury score [file 12906_2024_4366_MOESM1_ESM.pdf]

## H&E staining

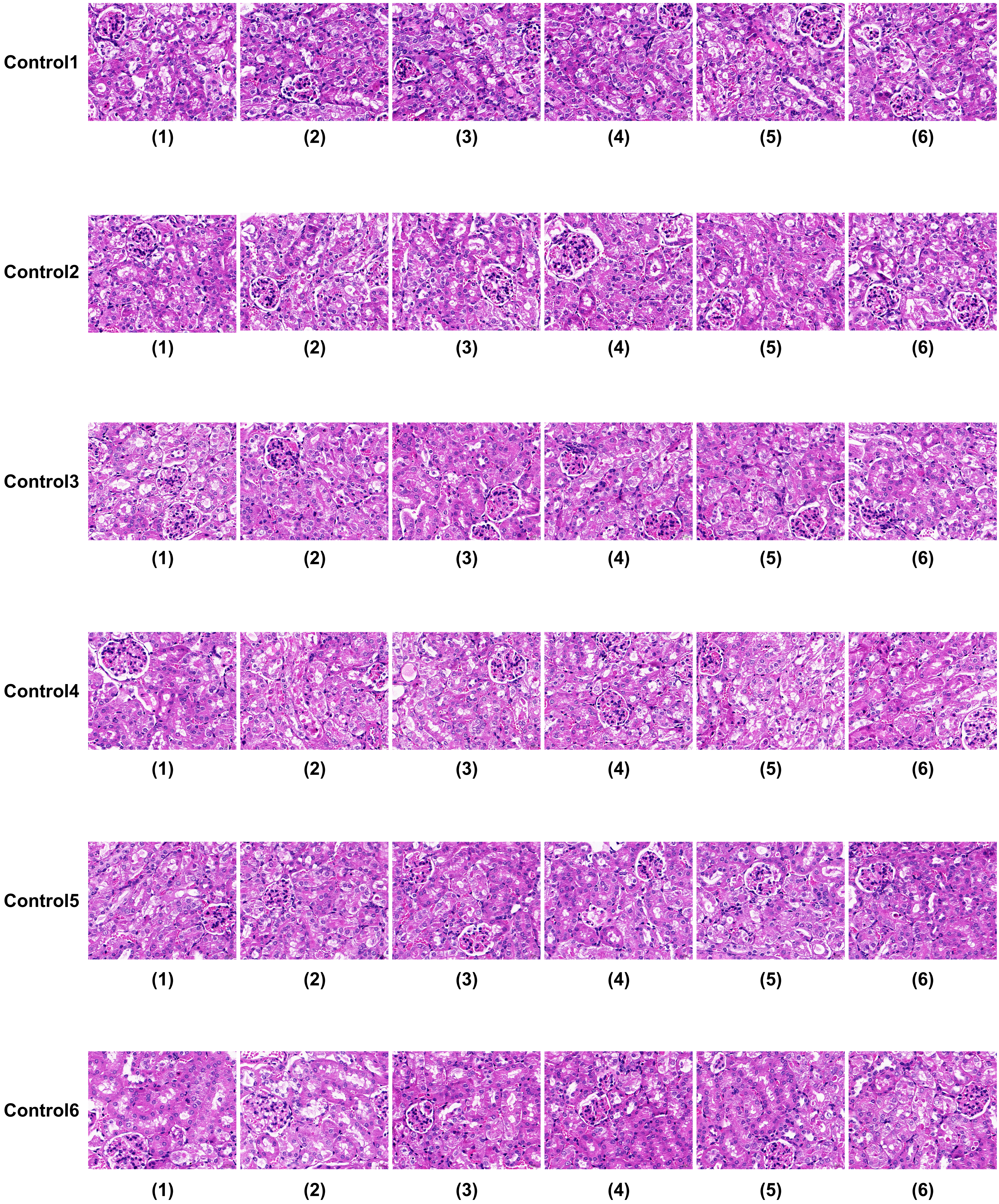
Control1
Control2
Control3
Control4
Control5
Control6
(1)
(2)
(3)
(4)
(5)
(6)

## H&E staining

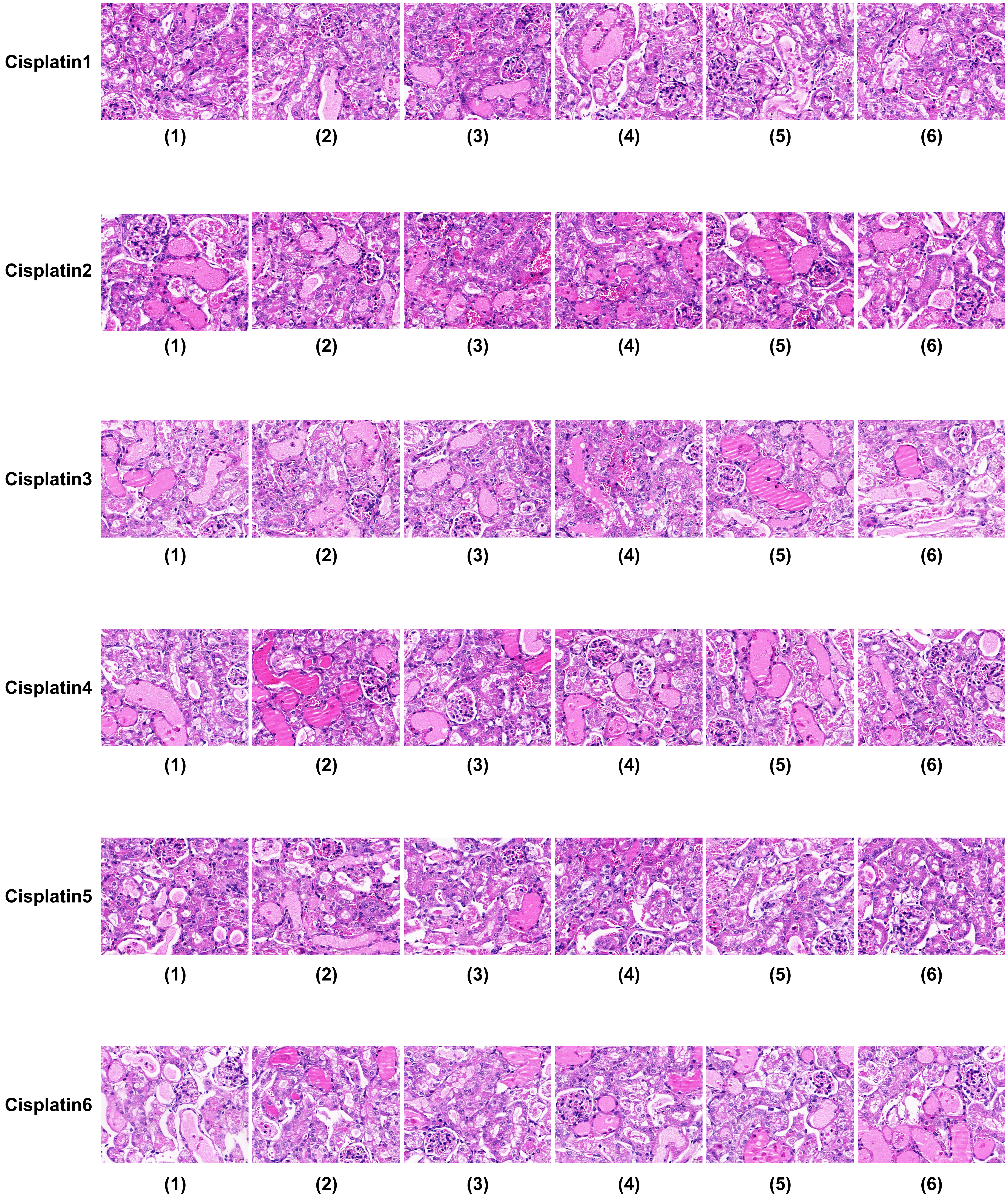
Cisplatin1
Cisplatin2
Cisplatin3
Cisplatin4
Cisplatin5
Cisplatin6
(1)
(2)
(3)
(4)
(5)
(6)

H&E staining

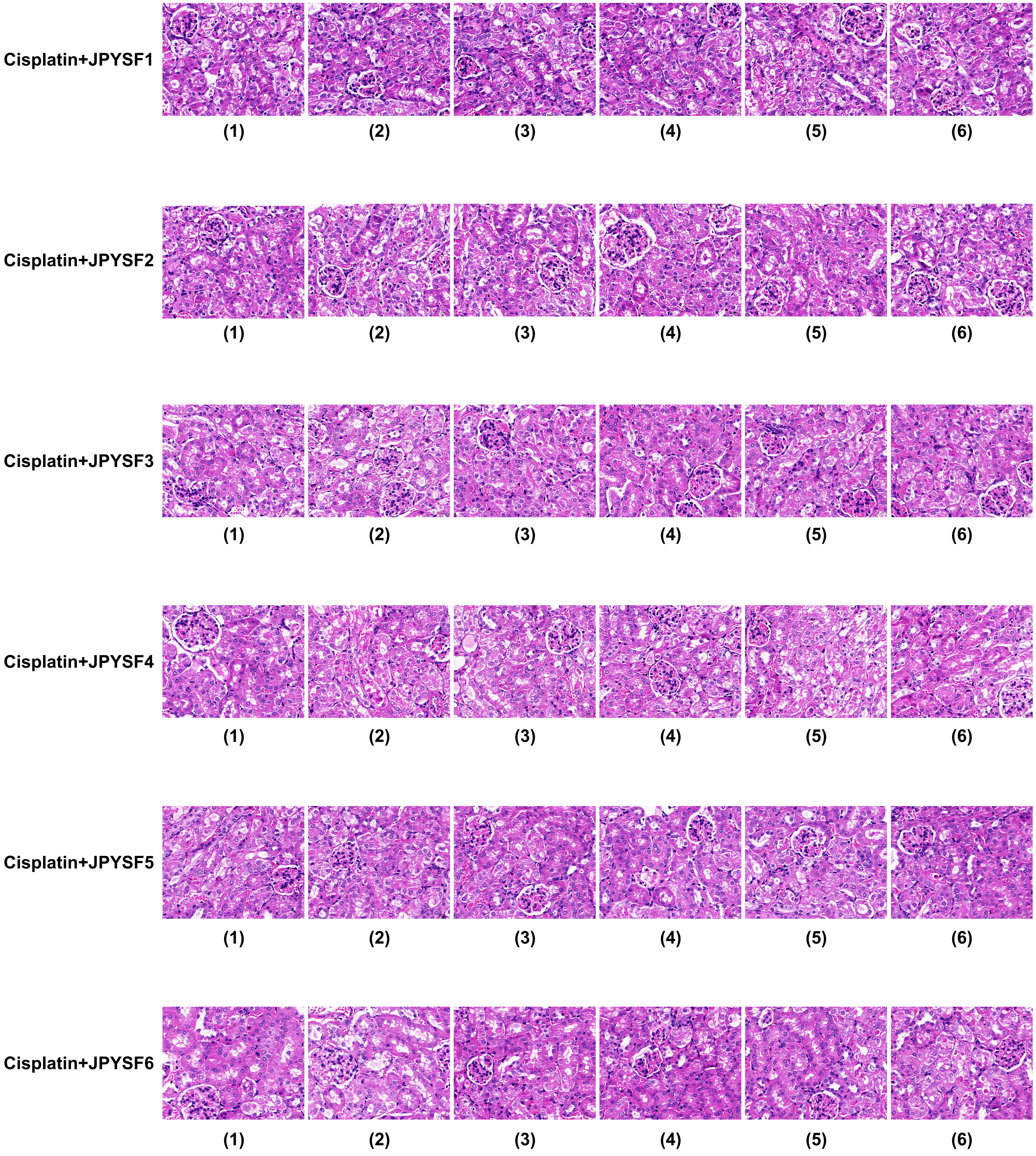

Cisplatin+JPYSF1
Cisplatin+JPYSF2
Cisplatin+JPYSF3
Cisplatin+JPYSF4
Cisplatin+JPYSF5
Cisplatin+JPYSF6
(1)
(2)
(3)
(4)
(5)
(6)
