# Supplementary material for: JianPiYiShen formula prevents cisplatin-induced acute kidney injury in mice by improving necroptosis through MAPK pathway
Source: BMC Complement Med Ther. 2024 Feb 24;24:101. doi: 10.1186/s12906-024-04366-9 (PMC10893720; doi:10.1186/s12906-024-04366-9)
Supplement: Supplementary file 3 — Supplementary Material 3: Western Blot raw data [file 12906_2024_4366_MOESM3_ESM.pdf]

Western blotting

Figure.1

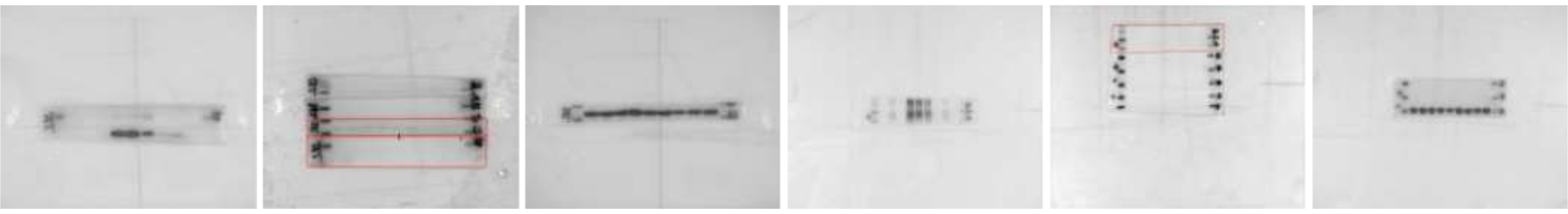

NGAL(1) | NGAL(2) | NGAL(gapdh) | KIM-1(1) | KIM-1(2) | KIM-1(gapdh)

The above images are raw data of WB results in Figure 1, and the image marked in red box corresponds to the WB bands in Figure 1.

Figure.2

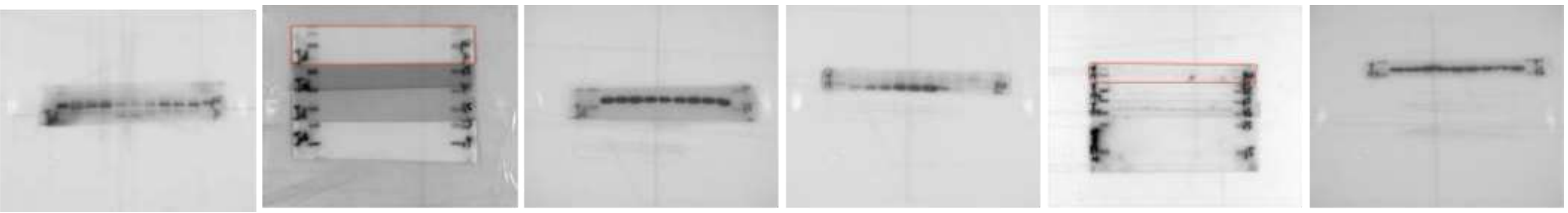

OPA1(1) | OPA1(2) | OPA1(gapdh) | DRP1(1) | DRP1(2) | DRP1(gapdh)

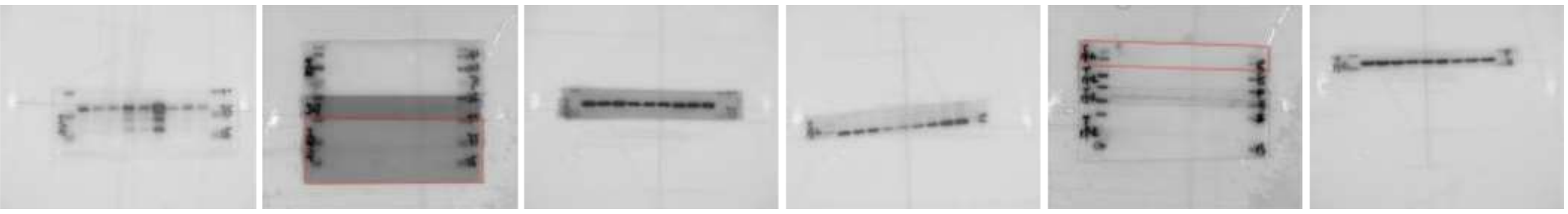

MFF(1) | MFF(2) | MFF(gapdh) | PGC-1α(1) | PGC-1α(2) | PGC-1α(gapdh)

The above images are raw data of WB results in Figure 2, and the image marked in red box corresponds to the WB bands in Figure 2.

Figure.3

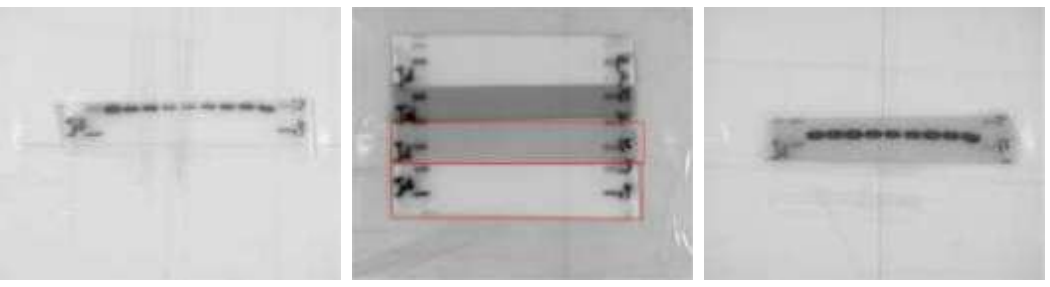

SOD1(1) | SOD1(2) | SOD1(gapdh)

The above images are raw data of WB results in Figure 3, and the image marked in red box corresponds to the WB bands in Figure 3.

Figure.4

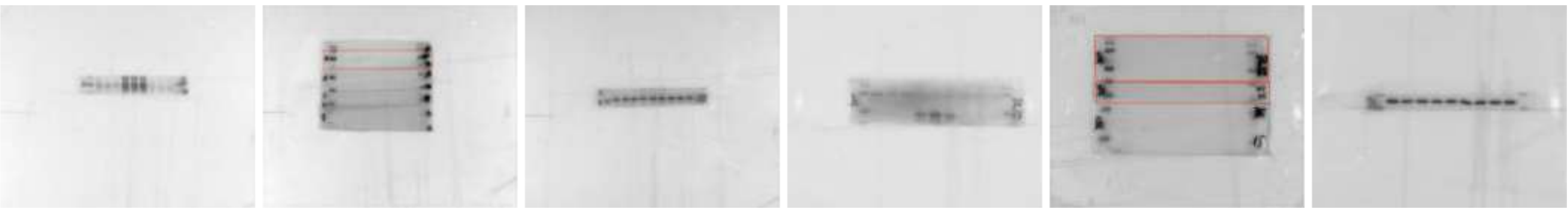

P-RIPK1(1) | P-RIPK1(2) | P-RIPK1(gapdh) | P-RIPK3(1) | P-RIPK3(2) | P-RIPK3(gapdh)

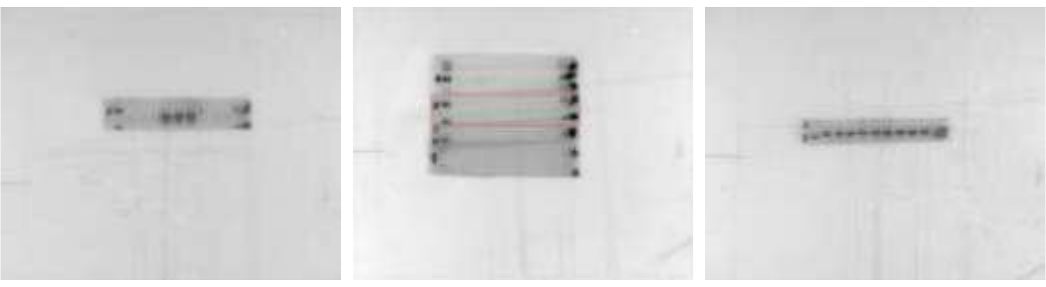

P-MLKL(1) | P-MLKL(2) | P-MLKL(gapdh)

The above images are raw data of WB results in Figure 4, and the image marked in red box corresponds to the WB bands in Figure 4.

Western blotting

Figure.5

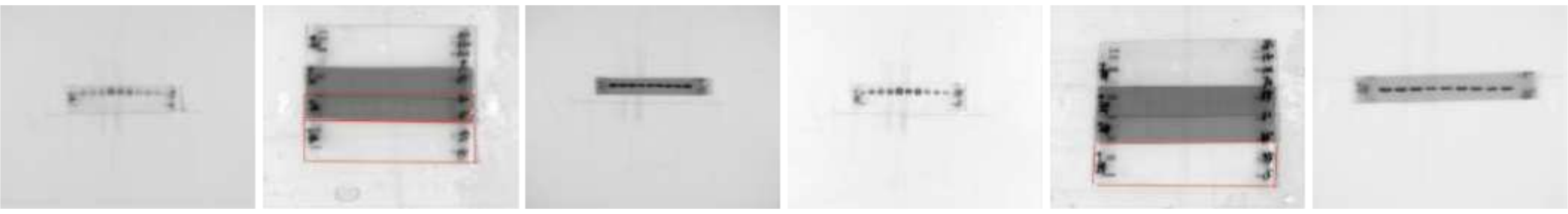

TNF-α(1) TNF-α(2) TNF-α(gapdh) IL-6(1) IL-6(2) IL-6(gapdh)

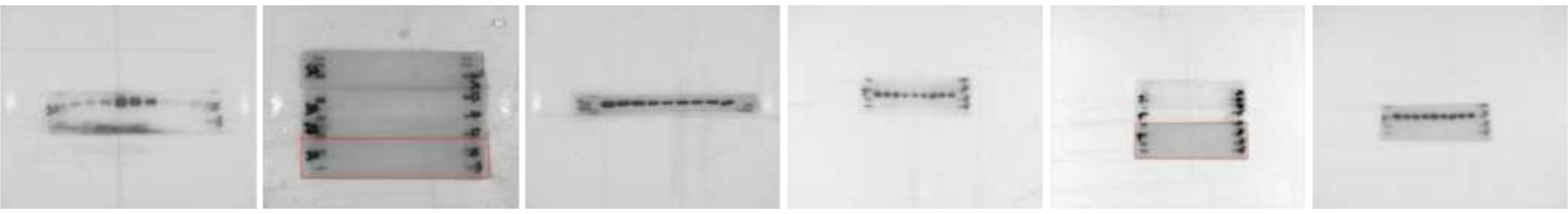

MCP-1(1) MCP-1(2) MCP-1(gapdh) IL-10(1) IL-10(2) IL-10(gapdh)

The above images are raw data of WB results in Figure 5, and the image marked in red box corresponds to the WB bands in Figure 5.

Figure.6

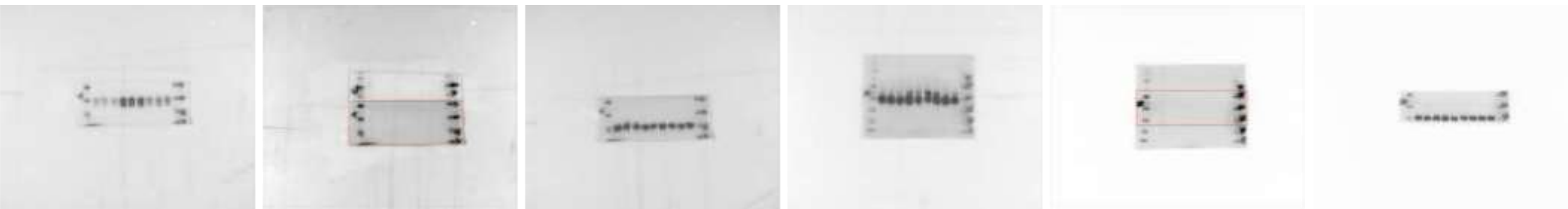

P-JNK(1) P-JNK(2) P-JNK(gapdh) JNK(1) JNK(2) JNK(gapdh)

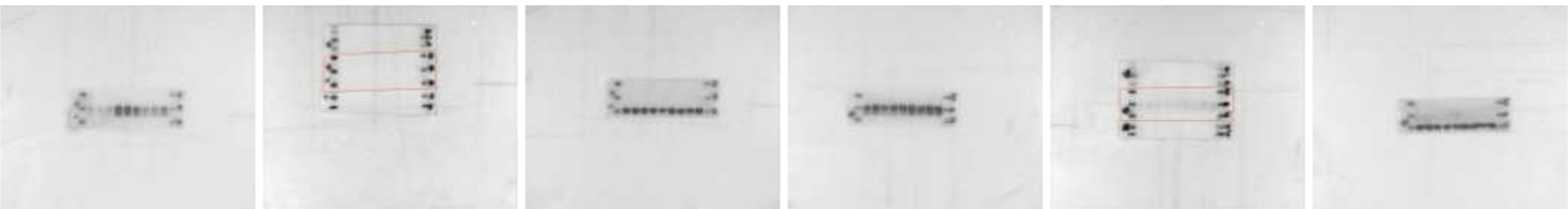

P-ERK(1) P-ERK(2) P-ERK(gapdh) ERK(1) ERK(2) ERK(gapdh)

The above images are raw data of WB results in Figure 6, and the image marked in red box corresponds to the WB bands in Figure 6.
